# Supplementary figures and images for: Deep momentum networks with market trend dynamics
Source: PLoS One. 2025 Sep 2;20(9):e0331391. doi: 10.1371/journal.pone.0331391 (PMC12404547; doi:10.1371/journal.pone.0331391)

S1 Fig. Annualized Sharpe ratios from five experiment iterations.

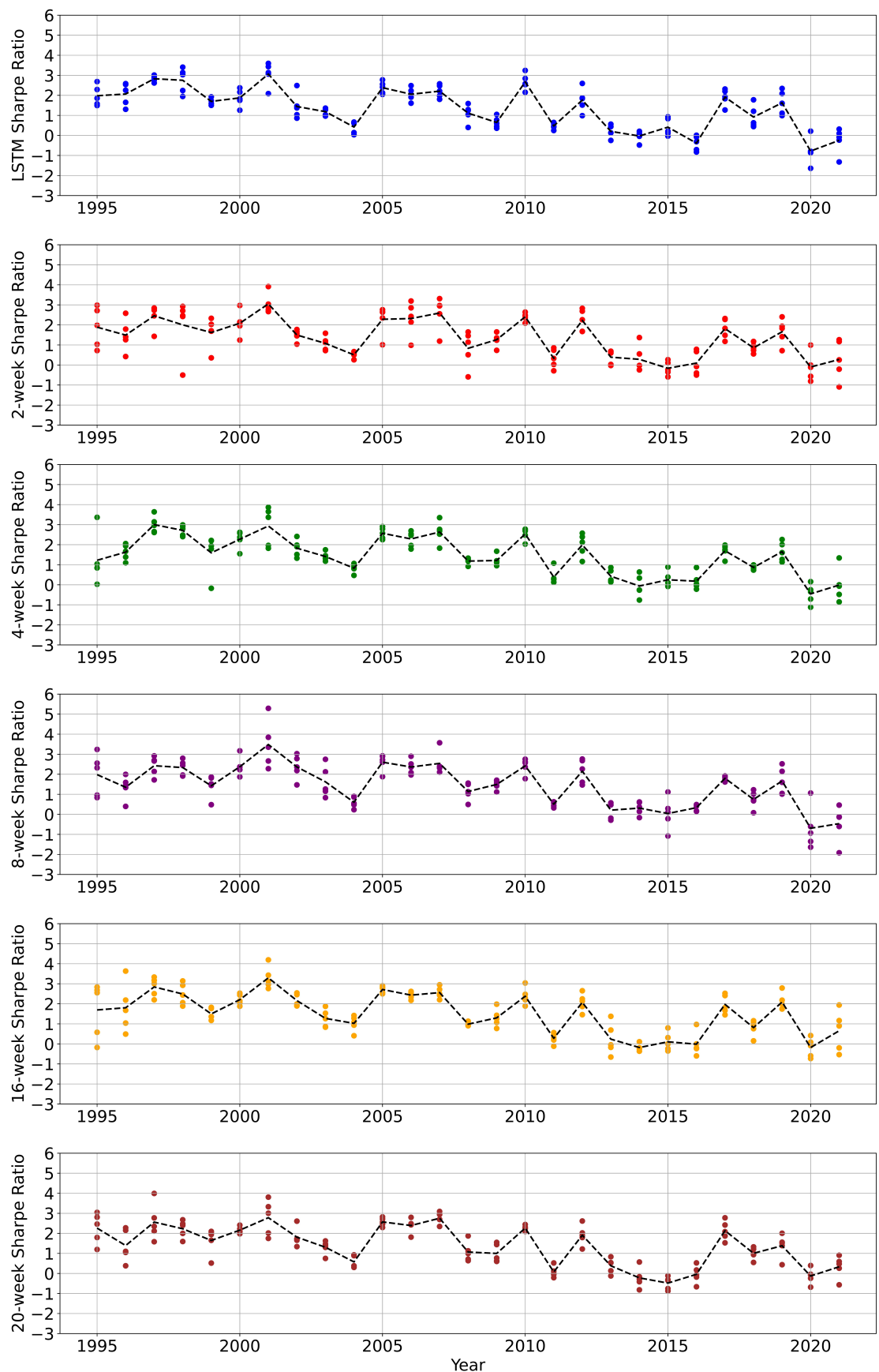

Supplement: S1 Fig — Each panel displays the results for a different model, allowing for a comparison of their performance consistency. (PDF) [file pone.0331391.s001.pdf]
