# Supplementary material for: Deep momentum networks with market trend dynamics
Source: PLoS One. 2025 Sep 2;20(9):e0331391. doi: 10.1371/journal.pone.0331391 (PMC12404547; doi:10.1371/journal.pone.0331391)

S1 Appendix. Cross-asset illustrations of MTDP-based position sizing.

This appendix graphically illustrates how the DMN, augmented with MTDP features, sizes positions and enhances cumulative returns across multiple asset classes.

Fig refA1 shows how incorporating the MTDP score influences position sizing and enhances performance over a 5-year backtest (2016–2020) for representative equity (S&P 500 index) and bond (Euro-Bobl) futures. The top panels display asset prices overlaid with MTDP signals (dashed lines), the middle panels show the resulting position sizes, and the bottom panels plot the cumulative returns. Traditional momentum strategies tend to underperform during correction or rebound phases, especially when fast and slow trend signals conflict. In contrast, the DMN leverages MTDP-based signals to dynamically adjust positions, improving robustness during such regime shifts.

A1. DMN-based trading strategies for the S&P 500 and Euro-Bobl futures (2016–2020).

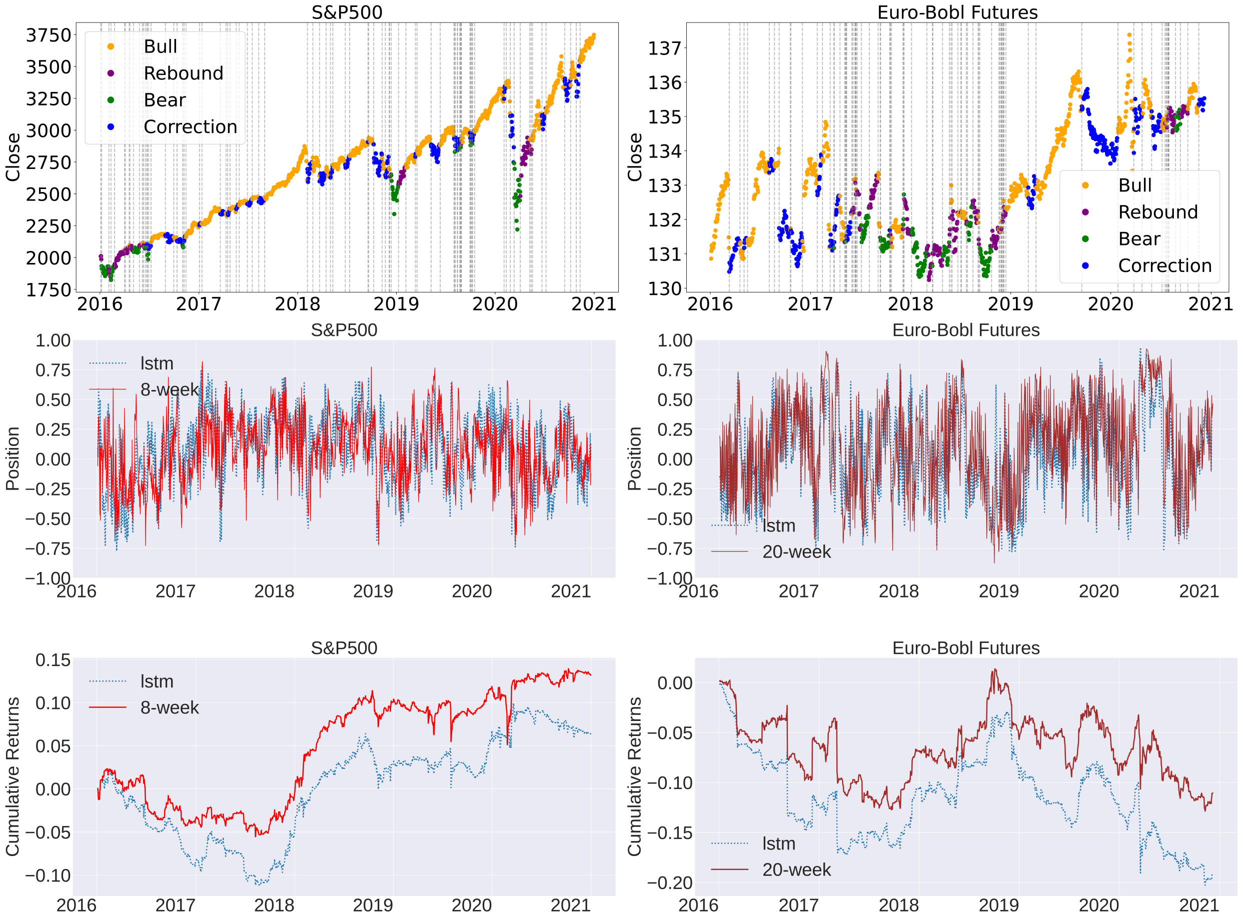

Fig A2–A5 compare cumulative returns under Setup 1 and Setup 2 for four representative asset classes: natural gas (commodities), DAX index (equities), 10-year US Treasury note (bonds), and GBP/CHF (foreign exchange). Notably, no single fast momentum window consistently dominates across all assets and market regimes. For instance, the 4- and 16-week signals perform best for natural gas in Setup 1, whereas the 8- and 20-week variants become more effective during volatile conditions in Setup 2. Similarly, different time windows emerge as optimal for DAX and Treasury futures depending on the scenario. These findings highlight the variability of market dynamics and suggest that adaptive or hybrid momentum strategies may be preferable in practice.

A2. Natural gas futures – Cumulative returns under Setup 1 and Setup 2.

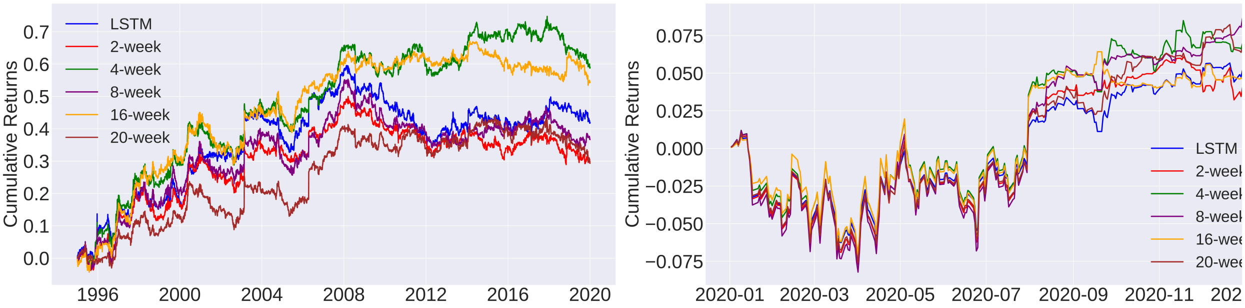

A3. DAX Index – Cumulative returns under Setup 1 and Setup 2.

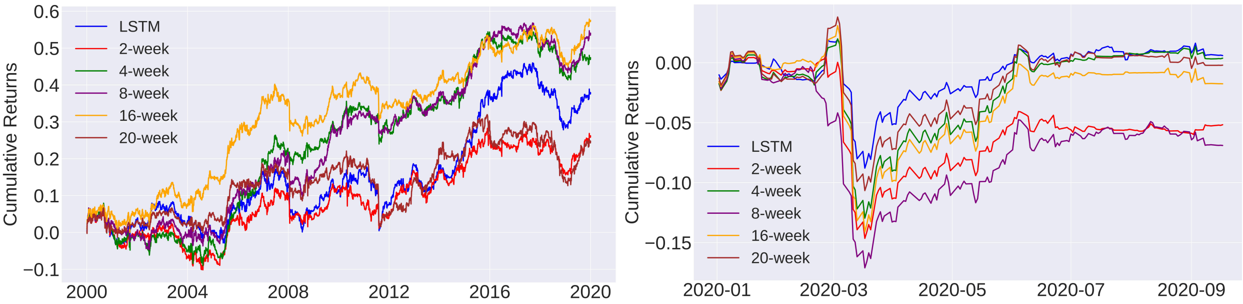

A4. 10-year US Treasury Note – Cumulative returns under Setup 1 and Setup 2.

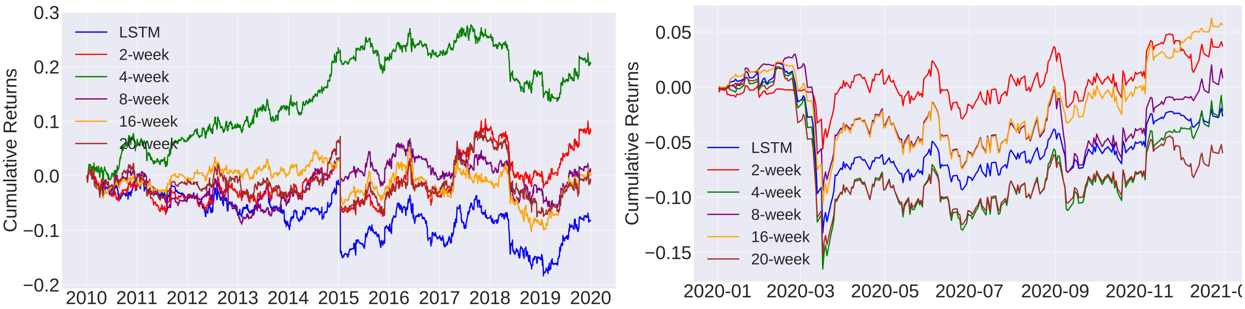

A5. British pound / Swiss franc pair – Cumulative returns under Setup 1 and Setup 2.

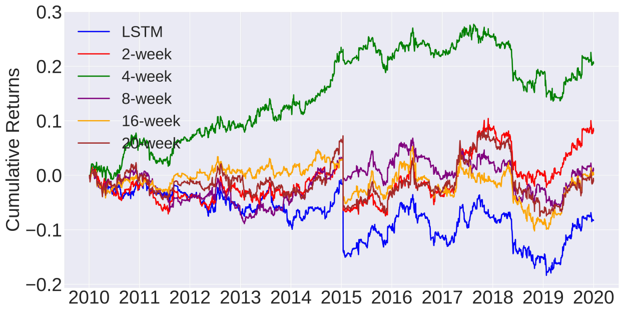

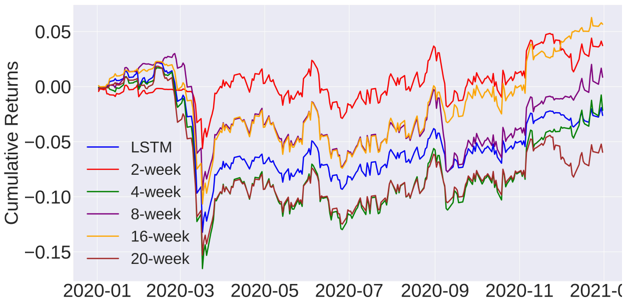

Supplement: S1 Appendix — Fig A1 shows how MTDP signals guide position sizing and enhance returns for equity and bond futures. Fig A2–A5 extend this to four asset classes across two scenarios, revealing that the best momentum window varies by asset and regime, highlighting the value of adaptive strategies. (PDF) [file pone.0331391.s002.pdf]
