# Supplementary material for: Deep momentum networks with market trend dynamics
Source: PLoS One. 2025 Sep 2;20(9):e0331391. doi: 10.1371/journal.pone.0331391 (PMC12404547; doi:10.1371/journal.pone.0331391)

S2 Appendix. Interpretability analysis via integrated gradients.

(a) Mathematical formulation of IG attribution and normalization.

Let $f : \mathbb{R}^d \to \mathbb{R}$ denote the model's scalar output (e.g., a position sizing), $\mathbf{x} \in \mathbb{R}^9$ the MTDP score vector, and $\mathbf{x}_0$ a baseline encoding the absence or neutrality of MTDP information. Following [1], IG assigns to feature i the path-integrated sensitivity along the straight line from $\mathbf{x}_0$ to $\mathbf{x}$:

$$\mathrm{IG}_i(\mathbf{x}, \mathbf{x}_0) = (x_i - x_{0,i}) \int_0^1 \frac{\partial f\big(\mathbf{x}_0 + \tau(\mathbf{x} - \mathbf{x}_0)\big)}{\partial x_i} \, d\tau. \tag{1}$$

In practice, we approximate the integral using a Riemann sum with $m = 50$ steps:

$$\mathrm{IG}_i(\mathbf{x}, \mathbf{x}_0) \approx (x_i - x_{0,i}) \cdot \frac{1}{m} \sum_{k=1}^{m} \left. \frac{\partial f(\mathbf{z})}{\partial x_i} \right|_{\mathbf{z} = \mathbf{x}_0 + \frac{k}{m}(\mathbf{x} - \mathbf{x}_0)}. \tag{2}$$

For each run r and rolling window w in Setups 1–3, the raw IG scores are converted to sign-preserving, L1-normalized relative importances across the nine MTDP components:

$$\widetilde{\mathrm{IG}}_i^{(r,w)} = \frac{\mathrm{IG}_i^{(r,w)}}{\sum_{j=1}^{9} \left| \mathrm{IG}_j^{(r,w)} \right|}, \tag{3}$$

which are then averaged over all runs $\mathcal{R}_s$ and windows $\mathcal{W}_s$ of the 4-week model for each Setup $s \in \{1, 2, 3\}$:

$$\overline{\mathrm{IG}}_i^{(\mathrm{4w,\,Setup}\ s)} = \frac{1}{|\mathcal{R}_s||\mathcal{W}_s|} \sum_{r \in \mathcal{R}_s} \sum_{w \in \mathcal{W}_s} \widetilde{\mathrm{IG}}_i^{(r,w)}. \tag{4}$$

References

[1] Sundararajan M, Taly A, Yan Q. Axiomatic attribution for deep networks. In: International conference on machine learning. PMLR; 2017. p. 3319–3328.

(b) IG-based feature importance visualization across all models and Setups.

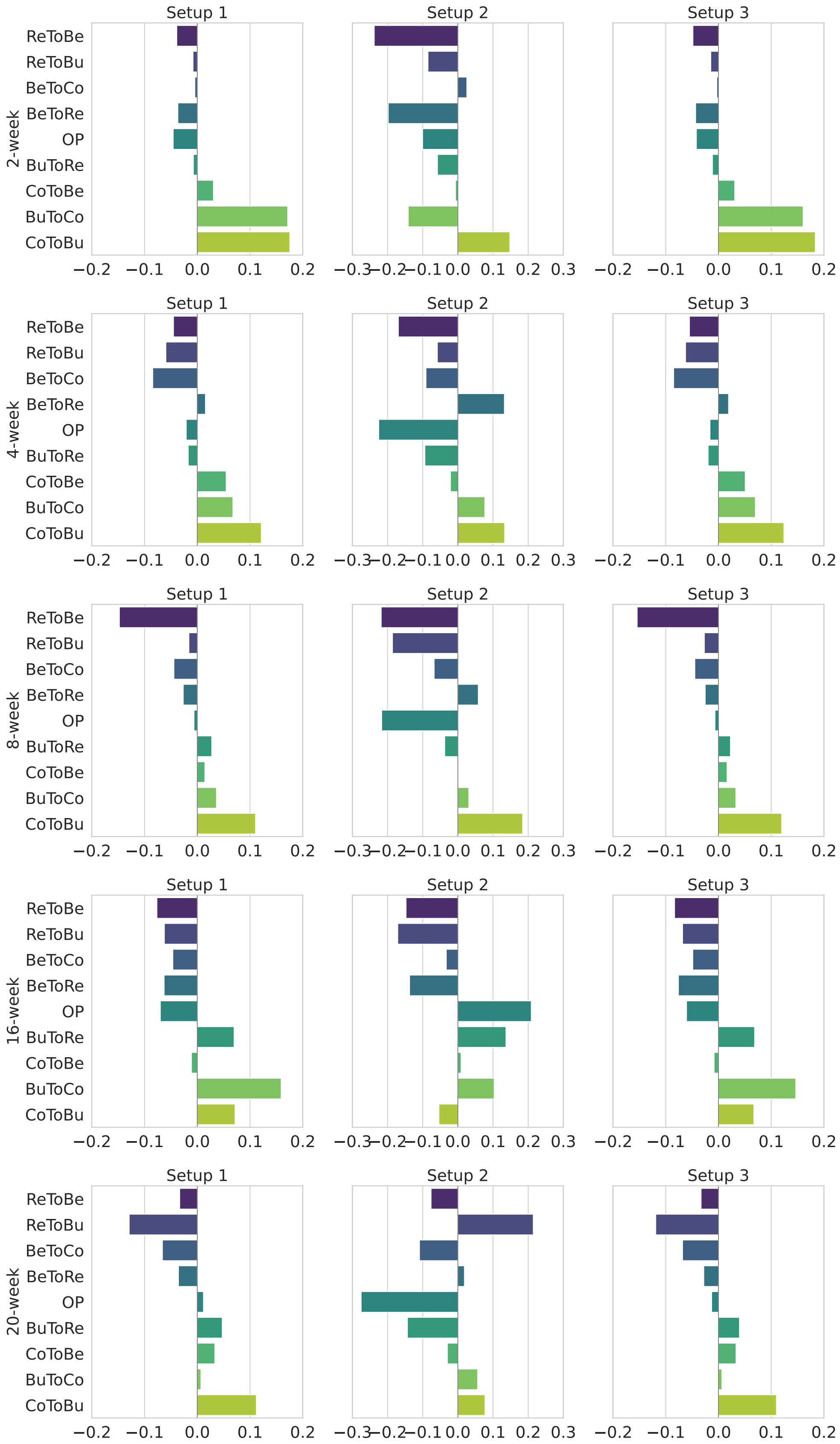

Supplement: S2 Appendix — (a) describes the mathematical formulation and normalization process used to compute IG-based feature importances for the MTDP scores. (b) shows the resulting average IG-based feature importances across all models and Setups. (PDF) [file pone.0331391.s003.pdf]
