# Supplementary material for: Deep momentum networks with market trend dynamics
Source: PLoS One. 2025 Sep 2;20(9):e0331391. doi: 10.1371/journal.pone.0331391 (PMC12404547; doi:10.1371/journal.pone.0331391)
Supplement: S1 Table — (PDF) [file pone.0331391.s004.pdf]

S1 Table. Asset-wise optimal XGBoost hyper-parameters for MTDP models using a 52-week look-back window and fast windows of 2, 4, 8, 16, 20 weeks across 99 assets.

	2-week			4-week			8-week			16-week			20-week		
Ticker	Depth	γ	Sub.	Depth	γ	Sub.	Depth	γ	Sub.	Depth	γ	Sub.	Depth	γ	Sub.
CBOE_VX	3	1.5	0.6	6	1.0	0.8	6	1.0	1.0	3	1.0	0.8	9	1.0	0.8
CME_AD	6	1.5	0.8	9	1.5	0.6	6	1.5	0.8	9	1.5	0.6	9	1.0	0.8
CME_AW	9	1.5	0.6	6	0.5	0.8	6	0.5	0.8	3	0.5	1.0	3	1.0	1.0
CME_B3	9	0.5	0.8	9	0.5	0.8	9	0.5	0.8	9	0.5	0.8	3	1.5	0.8
CME_BO	9	1.5	0.6	9	1.0	0.6	6	1.5	0.6	9	1.5	0.8	9	1.5	0.6
CME_BP	9	1.5	0.8	6	1.0	0.8	9	1.5	0.6	9	1.5	0.6	6	1.5	0.6
CME_BR	6	0.5	0.8	6	1.0	0.8	6	0.5	1.0	9	1.0	0.8	6	1.0	0.8
CME_BZ	9	1.0	0.6	3	1.0	1.0	3	0.5	0.6	6	1.0	1.0	3	0.5	0.8
CME_C	9	1.0	1.0	6	1.5	0.6	9	1.5	0.8	9	1.0	0.6	9	1.5	0.6
CME_CD	9	1.0	0.8	6	1.0	0.6	9	1.0	1.0	9	1.0	0.6	9	0.5	0.6
CME_CL	9	1.5	0.8	6	1.5	0.8	9	1.5	0.8	6	1.5	0.8	9	1.5	0.8
CME_EC	9	0.5	0.8	6	1.5	0.8	9	0.5	0.8	6	1.0	1.0	9	1.5	0.8
CME_EH	9	1.5	1.0	3	1.5	1.0	3	1.0	1.0	6	1.0	0.6	6	0.5	1.0
CME_ES	6	1.5	0.6	6	1.5	0.8	6	0.5	0.8	9	0.5	0.8	6	0.5	0.8
CME_FC	9	1.0	0.8	9	1.0	0.8	9	1.0	0.8	6	1.5	0.6	9	1.0	1.0
CME_FV	6	0.5	0.8	9	1.5	0.8	9	1.5	0.8	9	1.5	0.8	9	1.0	0.8
CME_GC	6	1.0	0.6	9	1.0	0.6	6	1.5	0.8	9	1.5	0.6	9	1.0	0.6
CME_HG	6	1.5	0.6	9	1.5	0.6	9	1.5	0.8	9	0.5	0.6	9	1.5	0.8
CME_HO	9	1.0	0.6	6	1.5	0.6	6	1.5	0.8	9	0.5	0.8	9	1.5	0.6
CME_JY	9	0.5	0.8	9	1.5	0.6	9	1.5	0.6	6	1.5	0.6	9	0.5	0.6
CME_KW	6	1.0	0.8	9	1.0	0.8	9	0.5	0.6	9	0.5	0.8	9	1.5	0.6
CME_LB	9	1.0	0.8	9	1.0	0.6	9	1.5	0.6	9	1.0	0.6	9	0.5	1.0
CME_LC	6	1.0	0.8	9	1.5	0.8	6	0.5	0.6	6	1.0	0.6	9	1.0	0.8
CME_LN	9	1.5	1.0	9	0.5	0.8	6	1.5	1.0	6	1.5	0.8	9	1.0	1.0
CME_MD	6	1.0	0.6	9	1.0	0.8	9	1.0	0.8	9	1.0	0.8	9	0.5	0.8
CME_MP	9	0.5	0.6	9	1.5	1.0	9	1.0	0.6	9	1.0	0.8	6	0.5	0.6
CME_NE	6	1.0	1.0	9	1.0	0.8	9	1.0	0.6	9	0.5	0.6	9	0.5	1.0
CME_NG	6	1.0	1.0	9	1.0	0.8	6	1.5	0.6	6	1.5	0.6	6	1.5	0.8
CME_NK	6	0.5	0.6	9	1.0	0.6	6	1.0	0.8	9	1.5	0.8	9	1.0	1.0
CME_NQ	9	1.5	0.8	6	0.5	0.8	9	0.5	0.6	9	0.5	0.8	6	1.0	0.6
CME_O	9	1.5	0.8	9	1.0	0.8	9	1.5	0.8	9	1.5	0.6	9	1.5	1.0
CME_PA	9	1.5	0.6	6	1.5	0.6	9	0.5	0.8	9	0.5	0.8	9	1.0	0.6
CME_PL	6	1.0	0.8	6	1.0	1.0	9	0.5	1.0	9	1.5	1.0	9	0.5	1.0
CME_RB	9	1.5	0.6	9	0.5	0.8	6	1.0	0.6	6	0.5	1.0	6	0.5	0.8
CME_RF	3	1.5	0.8	9	0.5	0.8	9	0.5	0.8	6	0.5	1.0	6	0.5	0.8
CME_RR	6	1.5	0.6	9	1.5	0.6	9	1.0	0.6	9	1.0	0.8	6	1.0	0.8
CME_RU	9	1.5	0.8	9	1.0	0.8	9	0.5	0.8	3	1.0	0.8	9	1.0	1.0
CME_S	6	1.0	0.6	9	1.0	0.8	9	1.0	0.8	6	1.0	1.0	3	0.5	0.6
CME_SF	9	1.0	0.8	9	1.5	0.8	6	1.0	1.0	3	1.5	0.6	6	0.5	0.6
CME_SI	6	1.0	0.6	6	1.5	0.6	9	0.5	0.8	6	1.0	0.6	9	1.0	0.6
CME_SM	6	0.5	0.6	6	0.5	0.8	9	1.5	0.8	9	1.0	0.6	6	1.5	1.0
CME_SP	6	0.5	0.6	9	1.0	0.6	9	1.5	0.8	9	1.0	0.6	9	1.5	0.8
CME_TU	6	1.0	1.0	9	0.5	0.6	6	1.0	0.8	6	0.5	0.8	3	1.0	1.0
CME_TY	6	0.5	0.8	6	1.0	0.6	6	0.5	1.0	6	1.0	0.6	9	1.0	0.6
CME_UL	3	1.5	1.0	6	0.5	1.0	6	0.5	1.0	3	0.5	1.0	6	1.5	1.0
CME_US	6	1.5	0.8	6	1.5	0.8	9	1.0	0.8	9	0.5	0.6	3	1.5	0.6
CME_W	6	1.0	0.6	6	1.0	0.6	9	1.0	0.8	6	1.5	0.6	6	0.5	0.8
CME_YM	6	0.5	0.8	6	0.5	1.0	6	0.5	1.0	9	1.0	1.0	6	0.5	1.0
EUREX_CONF	6	1.0	0.8	6	0.5	1.0	9	0.5	1.0	9	0.5	0.8	3	0.5	0.8
EUREX_FBTP	3	0.5	0.6	9	1.5	0.8	3	1.0	1.0	3	0.5	0.8	9	0.5	1.0
EUREX_FBTS	3	1.0	0.8	9	1.0	0.8	6	1.5	1.0	3	0.5	1.0	6	0.5	0.6
EUREX_FDAX	6	0.5	0.6	9	0.5	1.0	6	0.5	0.8	6	1.0	0.8	9	0.5	1.0
EUREX_FESX	3	1.0	0.8	9	1.0	1.0	6	1.0	1.0	9	0.5	0.8	9	0.5	1.0

Continued on next page

Ticker	Depth	γ	Sub.	Depth	γ	Sub.	Depth	γ	Sub.	Depth	γ	Sub.	Depth	γ	Sub.
EUREX_FGBM	9	1.5	0.8	9	0.5	0.8	6	0.5	0.8	6	1.5	0.8	6	0.5	1.0
EUREX_FGBX	6	1.5	0.6	6	1.0	1.0	6	1.0	1.0	3	1.0	1.0	9	1.0	1.0
EUREX_FOAT	6	1.0	1.0	3	0.5	1.0	9	1.0	0.8	3	0.5	0.8	9	0.5	1.0
EUREX_FRDX	6	0.5	0.8	6	0.5	0.8	6	1.0	1.0	3	0.5	1.0	9	1.0	1.0
EUREX_FSLI	6	0.5	0.8	9	0.5	1.0	9	1.5	1.0	3	0.5	0.8	6	0.5	0.8
EUREX_FSMI	9	0.5	1.0	6	0.5	0.8	9	0.5	1.0	9	0.5	1.0	3	0.5	0.8
EUREX_FSMM	6	1.5	1.0	6	0.5	0.8	9	0.5	1.0	6	1.5	1.0	3	0.5	1.0
EUREX_FSTX	9	1.0	0.8	9	0.5	0.8	6	0.5	0.8	6	0.5	1.0	6	0.5	1.0
EUREX_FTDX	6	0.5	0.6	6	1.0	0.8	3	0.5	1.0	3	0.5	1.0	9	1.0	0.8
EUREX_FVS	9	1.5	0.8	9	0.5	0.8	3	1.0	1.0	3	0.5	1.0	9	0.5	0.6
ICE_AR	6	1.5	1.0	6	0.5	0.8	6	0.5	1.0	3	0.5	0.6	9	1.5	0.8
ICE_B	9	1.0	0.8	6	1.5	0.6	6	0.5	0.8	6	1.0	0.8	3	1.5	0.8
ICE_BPB	6	1.0	0.8	6	1.0	0.8	9	0.5	0.6	3	0.5	1.0	6	0.5	1.0
ICE_C	6	1.5	0.8	6	1.5	1.0	6	1.5	1.0	9	1.5	0.6	3	1.5	0.8
ICE_CC	9	1.0	0.6	9	0.5	1.0	9	0.5	0.6	9	1.5	0.8	9	1.5	0.6
ICE_CEU	9	1.5	0.8	6	1.0	1.0	6	1.0	1.0	3	0.5	1.0	3	0.5	1.0
ICE_CT	6	1.5	1.0	6	1.0	0.6	9	1.0	0.8	9	0.5	0.8	9	1.0	0.6
ICE_DX	6	0.5	0.6	9	1.0	0.6	6	1.0	1.0	9	1.5	0.8	9	1.5	1.0
ICE_G	9	1.0	0.6	9	1.0	0.8	6	0.5	0.8	6	1.0	0.6	9	0.5	0.8
ICE_GER	6	0.5	0.8	9	0.5	0.8	9	0.5	1.0	3	0.5	0.8	9	0.5	1.0
ICE_GNM	3	0.5	1.0	6	0.5	0.8	6	1.5	1.0	3	0.5	0.8	6	0.5	1.0
ICE_KC	6	0.5	0.6	9	1.0	0.6	9	1.5	0.8	6	1.5	0.6	9	1.0	0.8
ICE_KRU	3	0.5	1.0	6	1.5	0.6	6	1.0	0.6	3	1.0	1.0	9	1.0	1.0
ICE_M	9	1.0	0.6	9	0.5	0.8	9	1.0	0.8	6	1.5	0.8	9	1.0	1.0
ICE_MP	3	0.5	0.6	9	1.0	0.6	9	0.5	0.8	6	1.0	0.8	9	0.5	0.6
ICE_N	9	1.5	0.6	6	0.5	0.8	6	1.0	0.8	3	0.5	0.8	6	1.0	1.0
ICE_NCF	6	1.5	0.8	6	0.5	0.6	9	0.5	1.0	6	0.5	1.0	9	1.0	1.0
ICE_NT	6	1.0	0.8	9	1.0	0.6	9	0.5	0.8	3	0.5	0.8	3	1.5	0.8
ICE_O	9	0.5	0.6	6	1.0	0.8	6	1.5	0.6	3	1.5	1.0	3	0.5	0.8
ICE_OJ	9	1.0	0.8	6	1.0	1.0	6	0.5	0.6	6	1.0	0.6	9	1.0	0.6
ICE_P	6	1.0	0.8	9	1.0	0.6	9	0.5	1.0	6	1.0	1.0	6	1.5	1.0
ICE_RS	6	0.5	0.6	9	1.0	0.6	6	0.5	0.6	9	1.0	0.8	6	0.5	0.8
ICE_SB	6	1.0	0.6	6	0.5	0.6	9	1.5	0.6	9	1.5	0.8	9	1.0	0.8
ICE_SS	6	1.0	0.6	9	0.5	1.0	6	0.5	0.8	6	0.5	1.0	6	0.5	0.8
ICE_SY	9	1.5	0.8	9	1.5	1.0	9	1.0	0.6	3	0.5	1.0	9	0.5	0.8
ICE_T	3	0.5	0.6	6	1.0	0.6	9	1.0	1.0	3	0.5	0.8	6	1.0	1.0
ICE_TIB	9	0.5	1.0	9	1.0	1.0	6	0.5	0.8	9	1.0	0.8	6	0.5	1.0
ICE_ZJ	9	0.5	0.8	9	1.0	1.0	6	1.0	0.6	6	0.5	1.0	9	0.5	1.0
ICE_ZR	6	1.5	0.6	6	1.0	0.8	9	1.0	0.8	9	1.0	1.0	9	0.5	0.8
LIFFE_C	9	1.5	0.6	6	1.5	0.6	6	1.0	1.0	9	1.5	0.8	9	1.0	0.6
LIFFE_FCE	6	0.5	1.0	6	1.5	0.8	6	0.5	0.6	9	1.5	1.0	6	0.5	0.8
LIFFE_R	6	0.5	0.6	9	1.5	0.8	9	1.0	1.0	6	0.5	0.8	3	0.5	0.8
LIFFE_RC	6	1.0	1.0	6	1.0	0.8	9	0.5	0.6	9	1.0	0.6	9	0.5	0.8
LIFFE_T	9	1.0	0.6	9	1.5	1.0	9	1.5	1.0	6	1.0	0.8	6	0.5	0.6
LIFFE_W	6	1.0	0.6	6	1.5	0.8	6	0.5	0.8	9	0.5	1.0	3	0.5	1.0
ODE_AB	9	1.5	0.6	9	0.5	0.8	6	1.0	0.8	9	1.0	0.8	3	0.5	0.6

Notes. Depth (= Max depth), γ (= Gamma), and Sub. (= Subsample) are the three hyper-parameters tuned in Table 3 (search ranges: Depth $\in \{3, 6, 9\}$, $\gamma \in \{0.5, 1.0, 1.5\}$, Sub. $\in \{0.6, 0.8, 1.0\}$). Ticker codes follow the <Exchange>_<Contract> convention; see S1 Appendix for the full contract list. Exchange prefixes: CBOE = CBOE Futures Exchange, CME = Chicago Mercantile Exchange, ICE = Intercontinental Exchange, EUREX = Eurex, LIFFE = London International Financial Futures and Options Exchange, ODE = Osaka Dojima Exchange.
