# Supplementary material for: Deep momentum networks with market trend dynamics
Source: PLoS One. 2025 Sep 2;20(9):e0331391. doi: 10.1371/journal.pone.0331391 (PMC12404547; doi:10.1371/journal.pone.0331391)
Supplement: S2 Table — (PDF) [file pone.0331391.s005.pdf]

S2 Table. Asset list and contract descriptions

Asset Class	Ticker	Description
Commodities	CME_AW	Dow Jones-UBS Commodity Index
Commodities	CME_B3	PJM Northern Illinois Hub 5 MW Peak Calendar-Month Real-Time LMP
Commodities	CME_BO	Soybean Oil
Commodities	CME_BZ	Brent Look-Alike
Commodities	CME_C	Corn
Commodities	CME_CL	WTI Crude Oil
Commodities	CME_EH	Ethanol
Commodities	CME_FC	Feeder Cattle
Commodities	CME_GC	Gold
Commodities	CME_HG	Copper
Commodities	CME_HO	Heating Oil
Commodities	CME_KW	KC HRW Wheat
Commodities	CME_LB	Lumber
Commodities	CME_LC	Live Cattle
Commodities	CME_LN	Lean Hogs
Commodities	CME_NG	Natural Gas
Commodities	CME_O	Oats
Commodities	CME_PA	Palladium
Commodities	CME_PL	Platinum
Commodities	CME_RB	Gasoline
Commodities	CME_RR	Rough Rice
Commodities	CME_S	Soybeans
Commodities	CME_SI	Silver
Commodities	CME_SM	Soybean Meal
Commodities	CME_W	Wheat
Commodities	ICE_B	Brent Crude Oil
Commodities	ICE_BPB	Endex Belgian Power Base Load
Commodities	ICE_C	EUA
Commodities	ICE_CC	Cocoa
Commodities	ICE_CEU	ECX EUAA
Commodities	ICE_CT	Cotton
Commodities	ICE_G	Gasoil
Commodities	ICE_GER	Endex German GASPOOL
Commodities	ICE_GNM	Endex German NCG
Commodities	ICE_KC	Coffee C
Commodities	ICE_M	UK Natural Gas
Commodities	ICE_N	NYH RBOB Gasoline
Commodities	ICE_NCF	Newcastle Coal
Commodities	ICE_O	Heating Oil
Commodities	ICE_OJ	Orange Juice
Commodities	ICE_P	UK Peak Electricity
Commodities	ICE_RS	Canola
Commodities	ICE_SB	Sugar No. 11
Commodities	ICE_T	WTI Crude
Commodities	ICE_TIB	WTI vs Brent Spread Option
Commodities	LIFFE_C	Cocoa
Commodities	LIFFE_RC	Coffee
Commodities	LIFFE_T	Feed Wheat
Commodities	LIFFE_W	White Sugar
Commodities	ODE_AB	Azuki Beans

Continued on next page

Asset Class	Ticker	Description
Equities	CBOE_VX	S&P 500 VIX
Equities	CME_ES	E-mini S&P 500 Index
Equities	CME_MD	S&P 400 MidCap Index
Equities	CME_NK	Nikkei 225 Index
Equities	CME_NQ	E-mini NASDAQ 100 Index
Equities	CME_SP	Full-Size S&P 500 Index
Equities	CME_YM	E-mini Dow Jones
Equities	EUREX_FDAX	DAX
Equities	EUREX_FESX	EURO STOXX 50 Index
Equities	EUREX_FRDX	RDX USD Index
Equities	EUREX_FSLI	SLI Swiss Leader Index
Equities	EUREX_FSMI	SMI
Equities	EUREX_FSMM	SMIM
Equities	EUREX_FSTX	STOXX Europe 50 Index
Equities	EUREX_FTDX	DivDAX
Equities	EUREX_FVS	VSTOXX
Equities	LIFFE_FCE	CAC40 Index
Fixed Income	CME_FV	5 Year Treasury Note
Fixed Income	CME_TU	2 Year Treasury Note
Fixed Income	CME_TY	10 Year Treasury Note
Fixed Income	CME_UL	Ultra Treasury Bond
Fixed Income	CME_US	30-Year Treasury Bond
Fixed Income	EUREX_CONF	Eurex CONF Swiss Confederation Bond
Fixed Income	EUREX_FBTP	Long-Term Euro-BTP
Fixed Income	EUREX_FBTS	Short-Term Euro-BTP
Fixed Income	EUREX_FGBM	Euro-Bobl
Fixed Income	EUREX_FGBX	Euro-Buxl
Fixed Income	EUREX_FOAT	Euro-OAT
Fixed Income	LIFFE_R	Long Gilt
Foreign Exchange	CME_AD	Australian Dollar
Foreign Exchange	CME_BP	British Pound
Foreign Exchange	CME_BR	Brazilian Real
Foreign Exchange	CME_CD	Canadian Dollar
Foreign Exchange	CME_EC	Euro FX
Foreign Exchange	CME_JY	Japanese Yen
Foreign Exchange	CME_MP	Mexican Peso
Foreign Exchange	CME_NE	New Zealand Dollar
Foreign Exchange	CME_RF	Euro/Swiss Franc
Foreign Exchange	CME_RU	Russian Ruble
Foreign Exchange	CME_SF	Swiss Franc
Foreign Exchange	ICE_AR	Australian Dollar/New Zealand Dollar
Foreign Exchange	ICE_DX	US Dollar Index
Foreign Exchange	ICE_KRU	Russian Ruble/US Dollar
Foreign Exchange	ICE_MP	Sm GBP/USD
Foreign Exchange	ICE_NT	US Dollar/Norwegian Krone
Foreign Exchange	ICE_SS	GBP/CHF
Foreign Exchange	ICE_SY	GBP/JPY
Foreign Exchange	ICE_ZJ	New Zealand Dollar/Japanese Yen
Foreign Exchange	ICE_ZR	US Dollar/South African Rand

Notes. Ticker codes follow the `<Exchange>_<Contract>` convention. Exchange prefixes: CBOE = CBOE Futures Exchange, CME = Chicago Mercantile Exchange, ICE = Intercontinental Exchange, EUREX = Eurex, LIFFE = London International Financial Futures and Options Exchange, ODE = Osaka Dojima Exchange.
