# Supplementary material for: Deep momentum networks with market trend dynamics
Source: PLoS One. 2025 Sep 2;20(9):e0331391. doi: 10.1371/journal.pone.0331391 (PMC12404547; doi:10.1371/journal.pone.0331391)
Supplement: Attachment — Submitted filename: Responses to Reviewer 2.pdf [file pone.0331391.s006.pdf]

Responses to Reviewer 2

We thank Reviewer 2 for their careful reading and valuable comments regarding the motivation, theoretical formulation, and contribution of our work.

Comment 1. *Clarification on Model Selection Criteria: The choice of five fast momentum look-back windows is reasonable, but further clarification on how these specific intervals were determined would enhance the methodological transparency.*

Response. We selected the five fast windows (2, 4, 8, 16, and 20 weeks) to follow an approximately exponential grid, balancing coverage of various time horizons while keeping the model parsimonious. As now explained in Section 2.3 (lines 99-–107), this approach extends prior work to a weekly frequency. We also clarify that intermediate windows (e.g., 6 and 12 weeks) were excluded for tractability, as preliminary tests showed they did not materially improve risk-adjusted outcomes while significantly increasing the hyper-parameter search space.

Comment 2. *Discussion of Model Robustness: The results suggest no universally optimal window size. A brief discussion on potential hybrid or adaptive models that dynamically adjust window lengths could be added in the conclusion or future work section.*

Response. We agree and have expanded the conclusion to outline a regime-aware hybrid design. Specifically, Section 6 (lines 494–501) now proposes a two-stage architecture that (i) employs a Hidden Semi-Markov Model to infer latent trend regimes and (ii) routes inputs through a Multi-Task Mixture-of-Experts network whose expert weights are modulated by the inferred regime probabilities. This adaptive approach enables smooth re-weighting among 2-, 4-, 8-, 16-, and 20-week experts and will be the focus of our follow-up study.

Comment 3. *Figures & Legends: Some figures (e.g., Figures 6 and 7) could benefit from more detailed captions to guide readers unfamiliar with the model architecture.*

Response. To improve clarity and manuscript flow, we have thoroughly revised our figures and moved the asset-specific examples to the Supporting Information (S1 Appendix).

- The figures referenced by the reviewer (formerly Figures 6 and 7) are now located in S1 Appendix as Figures A1–A5.
- All captions have been rewritten to be self-contained. For example, Figure A1's title now specifies the assets and timeframe ("DMN-based trading strategies for the S&P 500 and Euro-Bobl futures (2016-–2020)"), while the captions for Figures A2–A5 clarify the exact asset and backtesting setup used in each plot.

This approach ensures each visual is self-explanatory, directly addressing the reviewer's concern.

Comment 4. Typos and Formatting: A few minor typographical issues (e.g., "Correaction" instead of "Correction" in Table 2) should be corrected.

Response. We performed a manuscript-wide proofreading pass and fixed all detected issues.
